# Roles of Nitric Oxide and Asymmetric Dimethylarginine in Pregnancy and Fetal Programming

**DOI:** 10.3390/ijms131114606

**Published:** 2012-11-09

**Authors:** Li-Tung Huang, Chih-Sung Hsieh, Kow-Aung Chang, You-Lin Tain

**Affiliations:** 1 Department of Pediatrics, Kaohsiung Chang Gung Memorial Hospital, Chang Gung University College of Medicine, Kaohsiung 800, Taiwan; E-Mail: huang_li@pie.com.tw; 2 Department of Traditional Chinese Medicine, Chang Gung University, Linkow 333, Taiwan; 3 Department of Pediatric Surgery, Pingtung Christian Hospital, Pingtung 900, Taiwan; E-Mail: yenmeichang@yahoo.com.tw; 4 Department of Nursing, MeiHo University, Pingtung 900, Taiwan; 5 Department of Anesthesiology, Kaohsiung Chang Gung Memorial Hospital, Chang Gung University College of Medicine, Kaohsiung 800, Taiwan; E-Mail: kowaung@gmail.com; 6 Center for Translational Research in Biomedical Sciences, Kaohsiung Chang Gung Memorial Hospital, Chang Gung University College of Medicine, Kaohsiung 800, Taiwan

**Keywords:** asymmetric dimethylarginine, Nitric oxide, nitrergic, fetal programming, placenta

## Abstract

Nitric oxide (NO) regulates placental blood flow and actively participates in trophoblast invasion and placental development. Asymmetric dimethylarginine (ADMA) can inhibit NO synthase, which generates NO. ADMA has been associated with uterine artery flow disturbances such as preeclampsia. Substantial experimental evidence has reliably supported the hypothesis that an adverse in utero environment plays a role in postnatal physiological and pathophysiological programming. Growing evidence suggests that the placental nitrergic system is involved in epigenetic fetal programming. In this review, we discuss the roles of NO and ADMA in normal and compromised pregnancies as well as the link between placental insufficiency and epigenetic fetal programming.

## 1. Introduction

Nitric oxide (NO), the main vasodilator in the placenta, is involved in the regulation of feto-placental vascular reactivity, placental bed vascular resistance, trophoblast invasion and apoptosis, and platelet adhesion and aggregation in the intervillous space [1–3]. Placental vascular development is a crucial process required for adequate fetal development [1,4]. Placenta insufficiencies such as in gestational diabetes mellitus (GDM), intrauterine growth restriction (IUGR), and preeclampsia are related to vascular dysfunction of the placenta [5].

Plasma asymmetric dimethylarginine (ADMA) is recognized as a biomarker of endothelial disorders, cardiovascular disorders, hypercholesterolemia, and stroke [6–8]. Maternal plasma ADMA levels are reduced in a normal pregnancy but increase as the gestational age increases [9,10] and is increased in compromised pregnancies such as those with preeclampsia [9,10].

The placenta plays a central role in fetal programming by directly regulating blood flow, transporter activity, fetal nutrient supply and fetal growth [11,12]. Epigenetic mechanisms play a critical role during placental maturation and development [13]. NO is a known epigenetic molecule that plays a role in epigenetic fetal programming [14,15].

It has been suggested that therapeutic agents, which target placental blood flow and vascular development, ameliorate fetal growth restriction [2,5]. Thus, manipulation of the ADMA-NO pathways may have a therapeutic potential to rescue placental blood flow and improve long-term outcomes in patients with placental insufficiencies.

## 2. Metabolisms of NO and ADMA

NO is synthesized in every cell type by nitric oxide synthases (NOSs), namely nitric oxide synthase 1 (NOS1 or nNOS), nitric oxide synthase 2 (NOS2 or iNOS), and nitric oxide synthase 3 (NOS3 or eNOS). NOS1 and NOS3 are considered constitutive NOS. NO is formed from its precursor, l-arginine, by a family of NOSs in the presence of oxygen and the cofactor tetrahydrobiopterin, with the production of l-citrulline. NO produced in endothelial cells relaxes vascular smooth muscle via the cyclic guanosine monophosphate-dependent pathway and also has a potent vasodilatation effect. The exchange of amino acids between the intracellular environment and the plasma is facilitated by specific transporter systems. NOS activities depend on the ability of endothelial cells to take up their specific substrate l-arginine via a variety of membrane transport systems. In human endothelial cells, these membrane transport systems include y^+^, y^+^ L, b^0,+^, and B^0,+^[7,16].

**Figure 1:**
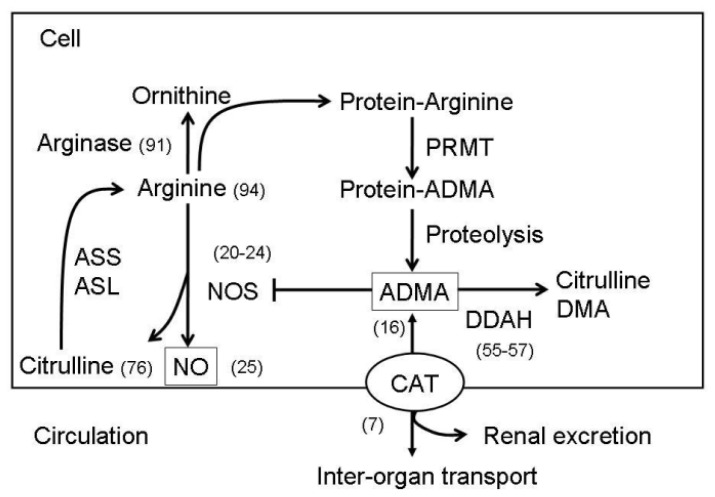
A schema showing the synthesis and metabolisms of ADMA-NO pathways. Protein-incorporated ADMA is formed by PRMTs. Free ADMA is then released after protein degradation. Free ADMA can be transported by CAT to move in or out of the cells. ADMA can be transported to major organs for ADMA degradation or excreted by the kidneys. ADMA is metabolized by DDAH to generate citrulline and DMA. Free ADMA can compete with arginine for NOS to generate NO and citrulline. Citrulline can be converted to arginine by ASS and ASL. In addition to NOS, arginine is the substrate for other metabolic pathways, such as arginase and protein synthesis. ASS, argininosuccinate synthetase; ASL, argininosuccinate lyase; CAT, cationic amino acid transporter; DMA, dimethylamine; NOS, nitric oxide synthese; ADMA, assymetric dimethylarginine; PRMT, protein arginine methyltransferase; DDAH, dimethylarginine dimethylaminohydrolase. Numbers in parentheses indicate the representative references.

ADMA is formed when arginine residues in proteins are methylated by the action of types I and II protein arginine methyltransferases (PRMT). ADMA is a competitive inhibitor of l-arginine for all 3 NOS isoforms. Approximately 20% of ADMA is excreted by the kidneys, whereas the other 80% is metabolized by two dimethylarginine dimethylaminohydrolases (DDAH-1 and -2) to l-citrulline and dimethylamine. System y^+^ transporters mediate ADMA uptake by neighboring cells and conform a family of proteins known as cationic amino acid transporters (CATs) that includes CAT-1, CAT-2A, CAT-2B, CAT-3, and CAT-4 isoforms [7]. The CATs are the main determinant of the ADMA distribution between the cytosol and the extracellular fluid. Intracellular ADMA can inhibit CAT, block NOS activity, and limit the cellular uptake of l-arginine, thereby contributing to oxidative stress and further NO biogenesis inhibition. The NO and ADMA synthesis and metabolism pathways are presented in Figure 1.

In preterm infants, plasma ADMA levels remain relatively constant during the first week of life but increase from 0.66 μM to 0.95 μM by the fourth week [17]. During childhood, ADMA levels slowly decrease from birth until around 25 years of age, with a mean decrease of 15 nM per year [18]. In adults, plasma ADMA concentrations tend to increase with age, with the mean concentration in the range of 0.4–0.6 μM [19].

## 3. Roles of NO and ADMA in Normal Pregnancies

During pregnancy, the mother and fetus have a complex anatomical and functional interaction. The placenta acts as the interface between the mother and the fetus and maintains fetal homoeostasis. The placenta originates from trophoblasts that differentiate into cytotrophoblasts and syncytiotrophoblasts, which form the primary villi. Villous cytotrophoblasts are specialized epithelial cells that anchor the fetus to the mother and establish blood flow to the placenta. The syncytiotrophoblasts, which comprise the transporting epithelium of the placenta, contain many transport proteins in the plasma membranes of both the mother and the fetus. These transport systems deliver nutrients to the fetus and provide a substrate for syncytiotrophoblast metabolism. Placental nutrient transport is dependent on vascular development; therefore, NO plays a critical role.

As pregnancy continues, NOS3 expression increases, primarily in the syncytiotrophoblasts [20]. In addition, NOS3 activity is present in the intermediate trophoblastic cells in the cell columns of the anchoring villi and in the trophoblastic cells at the implantation site [21]. On the other hand, NOS2 activity increases throughout pregnancy that peaks around mid-gestation [22]. Suzuki *et al.* proposed that NO plays a supportive role in promoting embryo survival [23]. Purcell *et al.* suggested that after implantation, both NOS3 and NOS2 might play a role in tissue remodeling, immunosuppression, and vasoregulation [24].

Plasma nitrates/nitrites have also been reported to be elevated in human pregnancy [25]. A study involving sheep showed that the pregnancy-associated increase in endothelium-dependent relaxation of the uterine arteries is predominantly regulated by the upregulation of NO release that results in decreased intracellular free calcium in the smooth muscle [26]. The human feto-placental vasculature lacks autonomic innervation, and therefore, NO confers autocrine and/or paracrine effects, playing an important role in the regulation of feto-placental blood flow. NO is the main vasodilator in the placenta and is involved feto-placental vascular reactivity regulation, placental bed vascular resistance, trophoblast invasion and apoptosis, and platelet adhesion and aggregation in the intervillous space [1].

Adequate morphology and function of the vascular tree are dependent on environmental cues such as blood flow, nutrient supply, and oxygen levels. Placenta vasculogenesis is the process of vessel formation from mesenchymal-derived hemangioblasts that differentiate into endothelial cells [27,28]. Differentiation of embryonic stem cells toward the endothelial lineage is an important step in vasculogenesis, and vascular endothelial growth factor (VEGF) is an important factor implicated in this process. In general, the initiation of vasculogenesis requires VEGF expression, which are effects mediated by NO [29]. The yolk sac plays a crucial role in embryo and placental vascular development. A study of mice yolk sacs showed that vasculogenesis is initiated by NO [30]. Furthermore, the spatio-temporal expression patterns of NOS2 and NOS3 were related to vasculogenesis in the yolk sac [30].

Angiogenesis, the formation of functional capillaries from pre-existing vasculature, helps drive villous development and differentiation [27]. In this process, single vessels are formed because endothelial precursor cells differentiate into endothelial cells. NO is a critical downstream mediator of potent angiogenic agents such as VEGF, basic fibroblast growth factor (FGF), and angiopoietin-1 [31]. The role of NO is more established in angiogenesis than in vasculogenesis [32].

The critical role of NO in angiogenesis has been shown in NOS3 knockout mice [33]. Likewise, NOS inhibition is accompanied by angiogenesis deficiencies as is exemplified by deficient vascular sprouting [34]. Interestingly, NO may also act upstream of angiogenic growth factors [35] because the effect of NO on VEGF production may be mediated through hypoxia-inducible factor-1.

Maternal plasma ADMA levels are reduced in a normal pregnancy but increase as the gestational age increases [9,10]. Holden *et al.* reported that the mean plasma ADMA concentratio in 20 nonpregnant women was 0.82 μmol/L [10]. The mean plasma ADMA concentration was 0.40 μmmol/L in 33 first-trimester pregnancies, 0.52 μmol/L in 50 second-trimester pregnancies, and 0.56 μmmol/L in 44 third- trimester pregnancies [10].

In early pregnancy, the reduction in ADMA and concomitant increase in NO may lead to hemodynamic adaptation, a higher need of organ perfusion in pregnancy, and uterine relaxation to allow for undisturbed intrauterine growth of the fetus. In late pregnancy, physiologically increased ADMA levels thus help prepare the uterine muscle fibers for the higher contractile activity that is necessary for successful delivery by antagonizing NO-induced uterine relaxation. This is reflected by the higher ADMA level after cesarean birth compared with vaginal birth [36]. After birth, ADMA levels fall, which may contribute to decreased NO production and bioavailability in neonatal vascular beds [36].

## 4. Roles of NO and ADMA in Compromised Pregnancies

NO regulates feto-placental vascular reactivity and placental blood flow. It has been suggested that placental angiogenesis abnormalities and angiogenic factor expression are associated with a variety of compromised pregnancies such as IUGR, GDM, and preeclampsia [37,38].

In an ewe model, circulating NO and its metabolites were elevated in pregnancies with multiple fetuses compared with singletons [39]. NOS3 can interact with the VEGF system and can enhance its production and function in placental tissues during early pregnancy [40]. In this context, placental expression of NOS3 is reduced in compromised pregnancies, including those of humans [41].

A normal pregnancy is accompanied by increasing metabolic activity of the placental mitochondria throughout gestation. The placenta thus generates reactive oxygen species (ROS) and reactive nitrogen species (RNS) in normal pregnancies and increased levels of the same in compromised pregnancies. In parallel, altered expressions of amino acid transporters in the placenta are also found in compromised pregnancies [1]. In this regard, Khullar *et al.* showed that NO^−^- and superoxide (O^2 −^)-derived free radicals impair placental syncytiotrophoblast amino acid uptake and increase Na^+^ permeability *in vitro*[42].

Due to their abundance in the placenta, superoxide and NO can interact to generate peroxynitrite (ONOO^−^), a potent pro-oxidant. Peroxynitrite formation will alter the placental NO level, which in turn will affect physiological function. The presence of nitrative stress was associated with diminished vascular reactivity of the fetal placenta, a situation that could be recapitulated *in vitro* by peroxynitrite treatment [43]. Roberts *et al.* found many nitrated proteins in the placenta [44]. Nitration of the placental proteins is found in normal pregnancies but at increased levels in pathologic pregnancies [45]. For instance, p38 mitogen-activated protein kinase could be nitrated with peroxynitrite at the tyrosine residues and could be associated with the loss of catalytic activity [45].

Recent studies have suggested that the plasma concentrations of ADMA may serve as a risk biomarker for endothelial dysfunction, hypercholesterolemia, preeclampsia, stroke, and cardiovascular disease [6]. Declining ADMA levels is observed in a normal pregnancy with increasing gestational age [9]. In compromised pregnancy such as preeclampsia, ADMA levels rise to levels greater than that seen in normal pregnancy [10]. Holden *et al.* showed that the mean plasma ADMA concentration of the 18 third-trimester preeclamptic subjects was 1.17 μmol/L, significantly higher than both the normotensive gestational age-matched and the nonpregnant control groups [10]. The association of abnormal uterine artery Doppler waveforms with high ADMA concentrations [46] in pregnancy supports the role of endogenous NOS inhibitors adversely affecting maternal vasodilation and blood pressure. Consistent with these findings, increased vasoconstriction of human stem villous feto-placental arteries [47] and increased ovine umbilical vascular resistance [48] are caused by an NOS inhibitor, N^G^-monomethyl-l-arginine. Suzuki *et al.* showed that N^G^-nitro-l-argininemethyl ester, an NOS inhibitor, caused apoptosis in the decidua, suggesting that NO in the decidua is essential to cell survival and the maintenance of uterine formation [49].

### 4.1. Preeclampsia

Preeclampsia is a major cause of fetal growth restriction, premature delivery, and maternal death worldwide. The underlying pathology of preeclampsia is presumably because of a relatively hypoxic or ischemic placenta. Preeclampsia is a disorder unique to pregnancy characterized by maternal hypertension, proteinuria, and edema.

In preeclampsia, the cytotrophoblast fails to adopt a vascular adhesion phenotype, resulting in compromised blood flow to the maternal-fetal interface. Through its unique angiogenic/vasculogenic properties, NO may be critical for cytotrophoblast endovascular invasion, an essential feature of normal placentation. Current evidence supports altered NO production in the feto-placental unit in preeclampsia [50]. In this regard, a reduced NO formation has been hypothesized to account for the abnormal placental perfusion in preeclampsia [51].

Increased ROS/RNS level contributes to endothelial dysfunction in the placenta and in the maternal vasculature and has been implicated as the pathophysiological feature of preeclampsia. An arginine deficiency documented in the preeclampsia placenta caused decreased NO and increased superoxide formation, leading to NO degradation and excess peroxynitrite formation [52]. Similarly, peroxynitrite formation is capable of attenuating vascular responses in the preeclampsia placenta [43]. In parallel, Myatt *et al.* showed elevated NOS3 expression in the placental vascular endothelium of preeclamptic women that was proposed to be a compensatory mechanism [53].

ADMA levels have been shown to increase even before the development of preeclampsia, suggesting that increased ADMA may be linked with the occurrence of preeclampsia in high-risk women [46]. In the study of Savvidou *et al.*, women who developed preeclampsia at a later stage had both bilateral uterine artery notches and impaired brachial artery flow-mediated dilation at 23–25 weeks’ gestation [46]. Since brachial artery flow-mediated dilation depends mainly on NO release, the increased ADMA levels and resultant endothelial dysfunction are thought to be attributable to the occurrence of preeclampsia. Placenta expresses high levels of DDAH-2 [54]. Anderssohn *et al.* showed decreased DDAH activity in the placenta from preeclamptic patients [55]. However, the association between DDAH polymorphisms and preeclampsia susceptibility remains inconclusive [56,57]. Whether impaired DDAH activity results in elevated ADMA levels that impair NO release and contributes to placental vascular dysfunction in preeclampsia awaits further elucidation.

### 4.2. Gestational Diabetes Mellitus

GDM is a syndrome characterized by glucose intolerance leading to maternal hyperglycemia, endothelial dysfunction, and abnormal regulation of vascular tone [58]. Placentas from GDM pregnancies are larger than normal [59] and show decreased formation of terminal villi and increased numbers of intermediate villi compared to those from normal pregnancies [60]. These vascular changes are likely to affect placental vascular resistance and vascular volume, leading to metabolic changes at the feto-placental microvascular and macrovascular endothelium [61].

Increased NO synthesis has also been reported in human placental veins and arteries from pregnancies with GDM [62]. GDM is associated with endothelial dysfunction characterized by an altered endothelial l-arginine/NO signaling pathway. In primary cultures of human umbilical cord endothelial cells (HUVECs) isolated from pregnancies with GDM, synthesis of NO [63,64] and l-arginine transport [64] and its intracellular concentration [61] are increased. Thus, altered placental vascular activity is characteristic of GDM and may be a consequence of a functional dissociation between NO synthesis and l-arginine uptake and/or bioavailability to the vascular endothelium and smooth muscle in the human placental circulation.

In addition, increased growth and vascularization in the GDM rat placenta are associated with higher levels of matrix metalloproteinase-2 (MMP-2) and MMP-9 [65], the activities of which are positively regulated by NO [66].

Akturk *et al.* investigated ADMA concentration in women with GDM and normal glucose tolerance during late pregnancy [67]. They found that ADMA concentration was elevated in women with GDM during late pregnancy and was positively correlated with the glucose levels in glucose challenge test [67].

### 4.3. Prenatal Malnutrition

Nutrient transfer across the placenta is essential for fetal growth and development. Prenatal malnutrition has important consequences in fetal growth and intrauterine programming. The placenta may act as a nutrient sensor, modifying nutrient and hormone availability to feto-placental tissues in relation to environmental challenges.

Placental NOS activities are 40%–45% lower in protein-deficient pigs than in protein-adequate pigs [68]. Maternal undernutrition in sheep decreases concentrations of arginine, citrulline, and polyamines in the maternal plasma, fetal plasma, and allantoic fluid [69]. In line with these findings, maternal undernutrition impairs NO-dependent vasodilation and increases arterial blood pressure in the ovine fetus [70].

The expression of angiogenic growth factors such as VEGF and basic FGF-2 as well as vascularity are altered in prenatally malnourished ewes [71]. Both VEGF [72] and FGF-2 [73] have been implicated in stimulating NO production in endothelium. On the other hand, NO can regulate both VEGF and FGF-2 expressions [31].

The labyrinthine layer, the site of feto-maternal interaction in the mouse placenta, contains both fetal and maternal blood vessels circulating independently of each other. Rutland *et al.* reported a reduction in labyrinthine blood vessel length and decreased expression of vascular endothelial adhesion molecules in the murine placenta in response to gestational protein malnutrition, suggesting that maternal nutrition alterations change placental vascular function [74].

We found increased plasma ADMA levels in the adult offspring from a 50% maternal caloric restriction rat model [75]. Since caloric restriction causes long-term somatic effects, we propose that increased plasma ADMA plays an important role in fetal programming.

### 4.4. Prenatal Glucocorticoid and Stress Exposure

Prenatal glucocorticoid therapy is used to prophylactically impede morbid symptoms associated with preterm delivery, such as respiratory distress syndrome and intraventricular hemorrhage. A recent medical practice survey showed that the proportion of women receiving antenatal corticosteroids had increased consistently over a seven-year period for those deliveries at 24–35 weeks and for those deliveries after 34 weeks [76]. It is well known that prenatal glucocorticoid and stress exposure lead to programming of hypothalamic-pituitary-adrenal function and behavior and have long-term effects on the offspring [77,78]. The effects of prenatal stress on fetal outcome are mediated in part by elevated fetal glucocorticoid exposure.

Altered placental glucocorticoid metabolism may influence placental efficiency by changing placental morphology, hormone synthesis, and transport physiology. VEGF is an important factor in the vasculogenesis process. Hewitt *et al.* first showed that prenatal glucocorticoid exposure induced fetal and placental growth restrictions by inhibiting placental VEGF expression and reducing placental vascularization [79]. VEGF-induced angiogenesis requires NO formation [80] derived from NOS3 activity [81]. In endothelial cells, VEGF induces NO synthesis via NOS3 through the activation of VEGFR-1 [82] and VEGFR-2 [83].

## 5. Placental Insufficiency and Developmental Programming

### 5.1. Role of the Placental Nitrergic System in Epigenetic Fetal Programming

Epigenetic modifications refer to stable and heritable gene expression changes that are not mediated by the alterations of the DNA sequence. Epigenetic mechanisms play a critical role during placental maturation and development [13]. Growing evidence indicates that NO exerts control over epigenetic mechanisms, including the function of histone deacetylase (HDAC) [14,15]. NO exerts its regulatory function on chromatin and gene expression *via* two chemical reactions: S-nitrosylation and tyrosine nitration [14]. Endothelial specialization and blood vessel formation are controlled by epigenetic mechanisms in the first stages of vascular development [84]. A study using HUVECs showed that the HDAC inhibitor, trichostatin A, reduced angiogenesis partially via regulation of NOS3-derived NO bioavalability [85]. Using a mouse cell line, Zeng *et al.* detected HDAC 3 in blood vessels during embryogenesis [86]. Together, NO might play a role in epigenetic fetal programming.

ADMA-related enzymes are also involved in compromised pregnancies and programming. Free ADMA levels are controlled by two counterbalancing pathways: PRMT I-related and DDAH-related. Several PRMTs have been associated with epigenetic regulation in which PRMTs act as histone methyltransferases or secondary co-regulators of transcription or facilitate mRNA splicing and stability [87]. At the organism level, several PRMTs seem to be important for development and may play an important role in organogenesis. Likewise, PRMT 5 was known to be a maternal detrimental factor of embyogenesis in fish [88]. However, the relationship between PRMTs and fetal programming remains poorly understood.

We and others have found that ADMA is involved in the fetal development of chronic kidney diseases and hypertension [7,89,90]. ADMA is implicated in the hypertension seen in spontaneous hypertensive rat [89]. Similarly, high prenatal salt intake in dams cause persistent hypertension in male offspring [90]. The observations that salt intake in dams cause adult male offspring increased serum ADMA concentrations and hypertension are in agreement with the fetal programming hypothesis [90]. In the study of Savvidou *et al.*[46], elevated plasma ADMA levels were seen in women who later on developed preeclampsia, suggesting the role of ADMA in fetal programming. However, the exact role of ADMA in fetal programming remains unclear and needs further studies.

### 5.2. Manipulations of the ADMA-NO Pathway to Prevent Compromised Pregnancies and Fetal Programming

Treatment of compromised pregnancy and fetal programming is currently limited to the management of complications. As stated above, the high morbidity and mortality of compromised pregnancy and the resulting fetal programming is likely due to an impaired ADMA-NO pathway. Thus, to preserve NO bioavailability, several therapeutic strategies have been used to either increase NO synthesis or decrease its breakdown. We recently reviewed that treatment with metformin, oral contraceptives, angiotensin-converting enzyme inhibitors, angiotensin receptor blockers, fenofibrate, folic acid, α-lipoic acid, melatonin, vitamin E, and *N*-acetylcysteine can lower plasma ADMA levels [16,91,92]; however, a specific ADMA-lowering agent remains unavailable.

l-arginine administration has been tested in many human diseases and experimental animals as a way to improve NO bioavailability. Some favorable effects of supplemental l-arginine on blood pressure and pregnancy outcomes were reported in patients with preeclampsia [93]. However, the data from human trials remain inconclusive. Since oral l-arginine treatment is hampered by pre-systemic elimination (e.g., hepatic and intestinal arginase) and l-citrulline can be converted to l-arginine, oral l-citrulline supplementation is a potential way to raise plasma l-arginine concentration and augment NO [94]. This thought is supported by our recent study that showed that maternal supplementation with l-citrulline increased renal NO bioavailability in the offspring of calorically restricted mothers [75]. In addition, l-arginine availability can be impaired because of consumption via other metabolic pathways (*i.e.*, arginase), reduced *de novo* synthesis, and impaired transport. For example, arginase inhibitor may be a potentially therapeutic approach to restoring l-arginine-NO to protect compromised pregnancies [95].

The impact of oxidative stress in pregnancy-related disorders and the use of antioxidants in perinatal medicine have been well reviewed elsewhere [96]. Importantly, the use of oxidative stress in interpreting developmental programming is an emerging hypothesis [97]. Even though some antioxidants have detrimental effects in experimental preeclampsia, antioxidant therapy to counter oxidative stress in human trials has failed to prevent preeclampsia [98]. It is clear that identification of the source of ROS and control of its production is a better strategy than non-selective use of antioxidants to prevent compromised pregnancy and fetal programming.

## 6. Conclusions

Placental nutrient transport is dependent on vascular development, which determines blood flow to the placenta. NO regulates placental blood flow. ADMA has been associated with uterine artery flow disturbances. Substantial experimental evidence has reliably supported the roles of NO and ADMA in normal pregnancy and placenta insufficiency as well as the link between placenta insufficiency and epigenetic fetal programming. With expanded knowledge on the mechanisms of the impaired ADMA-NO pathway, additional targets for therapeutic intervention will be identified. A multifaceted approach to restoring NO bioavailability will be required to prevent compromised pregnancies and fetal programming.

## Abbreviations

ADMA: Asymmetric dimethylarginine
CAT: Cationic amino acid transporters
DDAH: Dimethylarginine dimethylaminohydrolase
FGF: Fibroblast growth factor
GDM: Gestational diabetes mellitus
HDAC: Histone deacetylase
HUVEC: Human umbilical cord endothelial cells
IUGR: Intrauterine growth restriction
MMP: Matrix metalloproteinases
NO: Nitric oxide
NOS1 or nNOS: Nitric oxide synthase 1
NOS2 or iNOS: Nitric oxide synthase 2
NOS3 or eNOS: Nitric oxide synthase 3
PRMT: Protein arginine methyltransferases
RNS: Reactive nitrogen species
ROS: Reactive oxygen species
VEGF: Vascular endothelial growth factor
